# Nrac, a Novel Nutritionally-Regulated Adipose and Cardiac-Enriched Gene

**DOI:** 10.1371/journal.pone.0046254

**Published:** 2012-09-27

**Authors:** Ren Zhang, Fayi Yao, Feng Gao, Abdul B. Abou-Samra

**Affiliations:** 1 Center for Molecular Medicine and Genetics and Endocrine Division, School of Medicine, Wayne State University, Detroit, Michigan, United States of America; 2 Department of Physics, Tianjin University, Tianjin, China; University of Texas Southwestern Medical Center, United States of America

## Abstract

Obesity increases the risk of multiple diseases, such as type 2 diabetes and coronary heart diseases, and therefore the current obesity epidemic poses a major public health issue. Therapeutic approaches are urgently needed to treat obesity as well as its complications. Plasma-membrane proteins with restricted tissue distributions are attractive drug targets, because of their accessibility to various drug delivery mechanisms and potentially alleviated side effects. To identify genes involved in metabolism, we performed RNA-Seq on fat in mice treated with a high-fat diet or fasting. Here we show that the gene A530016L24Rik (human ortholog C14orf180), named Nrac, is a novel nutritionally-regulated adipose and cardiac-enriched gene. *Nrac* is expressed specifically and abundantly in fat and the heart. Both fasting and obesity reduced *Nrac* expression in white adipose tissue, and fasting reduced its expression in brown fat. Nrac is localized to the plasma membrane, and highly induced during adipocyte differentiation. Nrac is therefore a novel adipocyte marker and has potential functions in metabolism.

## Introduction

Obesity is a chronic disease that is becoming one of the most significant contributors to ill health [1]. A large body of evidence indicates that obesity is associated with an increased risk of multiple diseases, such as type 2 diabetes, hypertension, coronary heart diseases, musculoskeletal diseases and mortality [2]. Obesity is prevalent in both developed and developing countries, in both adults and children [1]. In the United States, between 1980 and 2002, obesity prevalence doubled in adults, and overweight prevalence tripled in children and adolescents. Now more than half of adult Americans are overweight or obese [3]. Similar trends were seen in other countries, such as Great Britain and China [4], [5], [6]. Clearly, we are in the midst of a global obesity epidemic.

Since the discovery of the obese gene *leptin* in 1994 [7], there has been an explosion in our knowledge to understanding the etiology of obesity and mechanisms underlying its various complications [8], [9], [10], [11], [12], [13], [14]. Adipose tissue mass increases in obesity; nevertheless, adipose tissue is no longer considered solely a passive depot for energy storage. In contrast, adipose tissue is in fact an active metabolic and endocrine organ with critical roles in regulating systemic physiology [13], [14], [15], [16], [17], [18].

Microarray technology has been routinely used to identify differential expression of genes in fat at different nutritional states, such as diet induced obesity and fasting [19], [20], [21]. However, microarray technology has some disadvantages, such as low sensitivity and limitation to examine only known genes. In contrast, RNA-seq (Whole Transcriptome Shotgun Sequencing), a new sequencing based technology, overcomes these shortcomings by being both sensitive and able to identify novel transcripts [22]. We therefore performed RNA-seq experiments on white adipose tissue (WAT) in mice treated with a high-fat diet (HFD) or fasting. A number of nutritionally regulated genes were identified (to publish elsewhere), and here we focus on the novel gene, A530016L24Rik, named Nrac (nutritionally-regulated adipose and cardiac-enriched). Being specific to fat and the heart, *Nrac* expression is reduced by both obesity and fasting in WAT. It is localized to the plasma membrane and highly induced during adipogenesis. Therefore, Nrac is a novel gene with potential functions in metabolism.

## Results and Discussion

### The Novel Gene Nrac and its Orthologs

**Figure 1:**
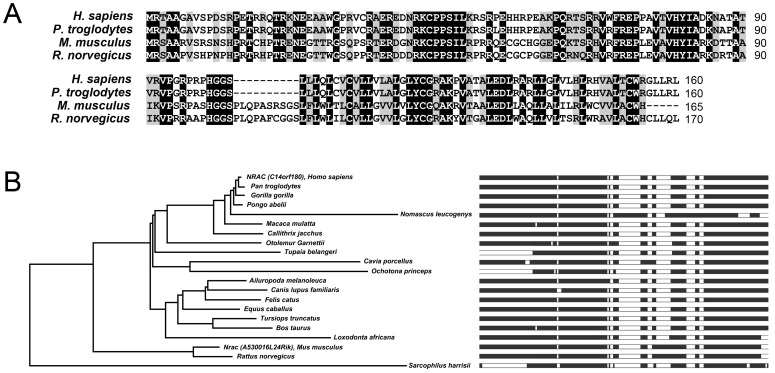
The Nrac protein sequence and orthologues. A) Alignments of Nrac protein sequences of *Homo sapiens, Pan troglodytes, Rattus norvegicus* and *Mus musculus*. B) Evolutionary tree of Nrac, based on the Ensembl Compara GeneTrees database. Refer to Materials for Nrac IDs from different species.

To comprehensively identify nutritionally regulated genes, we performed RNA-seq experiments on white adipose tissue, in mice treated with 3-month HFD or 24-hour fasting, together with controls. Here, we focus on the analysis of the novel gene *Nrac*, which was one of the identified genes that were sensitive to nutritional stimulation. The symbol for mouse Nrac is A530016L24Rik, and its human homolog is C14ORF180 (Table 1). Human and mouse Nrac protein sequences have 160 and 165 amino acids, respectively, and their alignment showed that 50% percent of residues were identical (Fig. 1A). The database of Ensembl Compara GeneTrees [23] identified 21 Nrac orthologs, all of which were from mammalian species (Fig. 1B). Using the human NRAC protein sequence, we performed Blast search against the database that contained all non-redundant protein sequences at NCBI, Swissprot and PIR. The Blast program was DELTA-BLAST (Domain Enhanced Lookup Time Accelerated BLAST), which is sensitive in detecting remote protein homologs [24]. In addition to the 21 Nrac orthologs in the Compara GeneTrees database, two protein sequences, from *Oreochromis niloticus* (the Nile Tilapia) and *Anolis carolinensis* (an arboreal lizard) also showed significant alignments. Therefore, Nrac appears to be evolutionarily conserved in mammals, and has orthologs in some other vertebrates.

**Table 1:** IDs of Nrac in databases.

	Mouse	Human
Name	Nrac	NRAC
Symbol	A530016L24Rik	C14ORF180
Chromosome	12	14
Location (bp)	113727659–113738138	105046056–105056183
RefSeq	NM_177039	NM_001008404
Ensembl	ENSMUSG00000043122	ENSG00000184601
Entrez	319942	400258
Uniprot	Q8BNX7	Q8N912

### Nrac Expression is Specific to Fat and Heart

**Figure 2:**
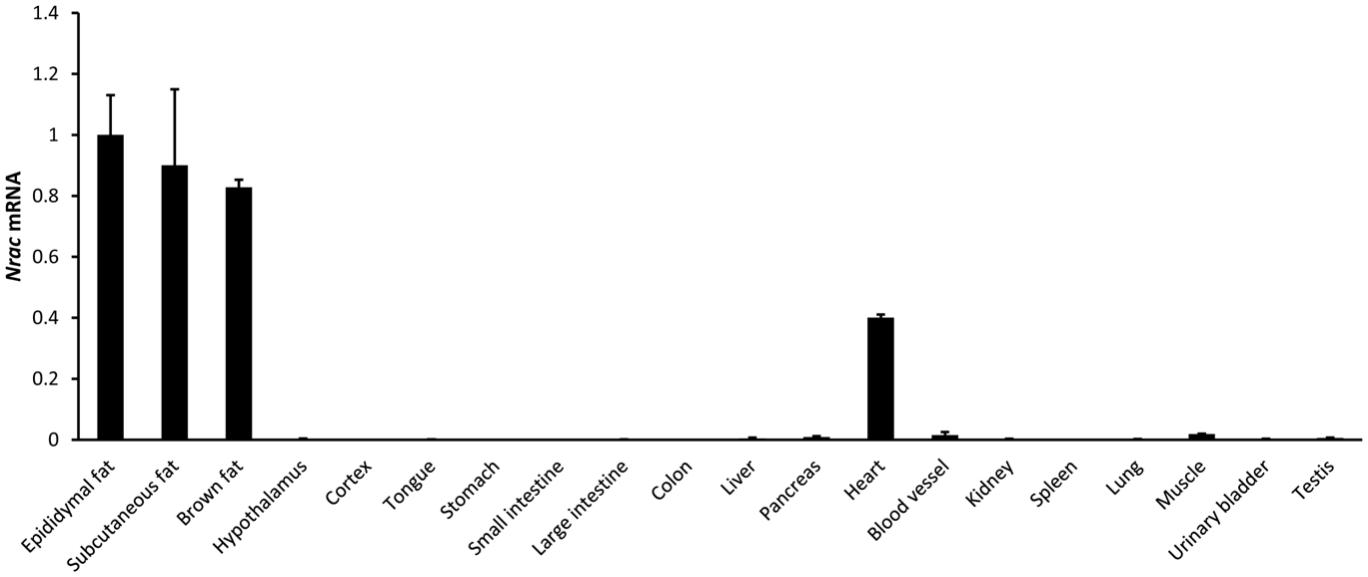
Nrac is specific to adipose tissues and the heart. *Nrac* mRNA distribution among different mouse tissues.

Tissue expression pattern for a novel gene can be informative in revealing its functions. We therefore examined the expression pattern of Nrac in various mouse tissues. Three male C57BL6 mice were used to dissect various tissues, including hypothalamus, cortex, tongue, stomach, small intestine, large intestine, colon, liver, pancreas, heart, blood vessel, kidney, spleen, lung, muscle, urinary bladder, testis, and fat. The fat tissues included epididymal fat, inguinal subcutaneous fat and brown fat (from scapular region). *Nrac* is abundantly expressed in heart and fat, including epididymal fat, subcutaneous fat and brown fat, and virtually non-existent in other tissues examined (Fig. 2). Therefore, Nrac expression is specific to fat and the heart.

### Nrac is Nutritionally Regulated

**Figure 3:**
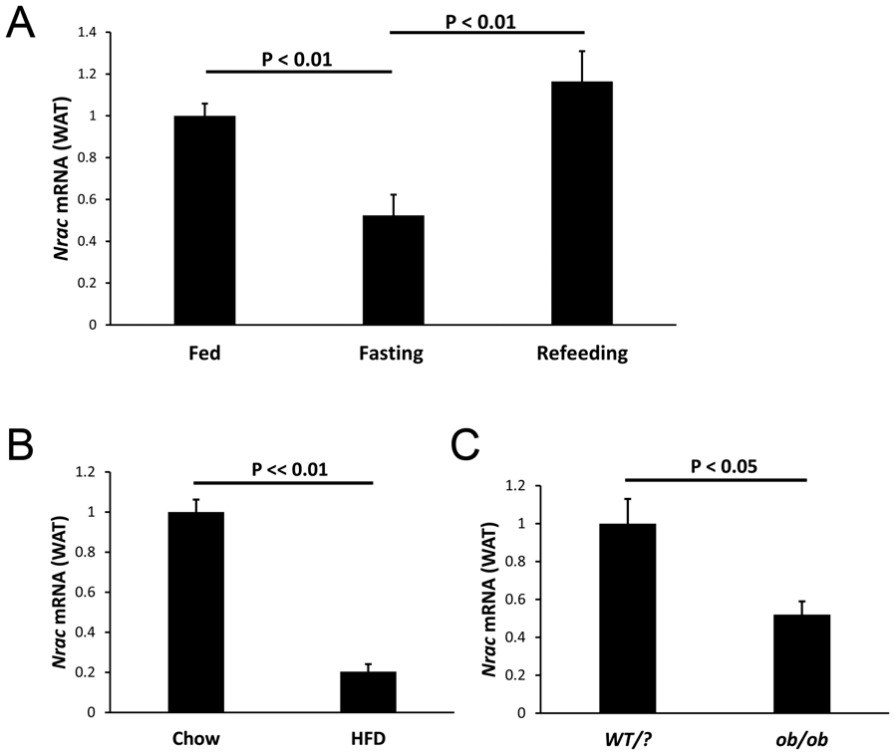
Nrac in white adipose tissue (WAT) by nutritional stimulation. A) *Nrac* is down regulated by 24-hour fasting, and normalized by 4-hour refeeding in WAT (epididymal fat). B) WAT *Nrac* is down regulated in diet induced obesity and in *ob*/*ob* mice. Data are represented as mean ± SEM.

**Figure 4:**
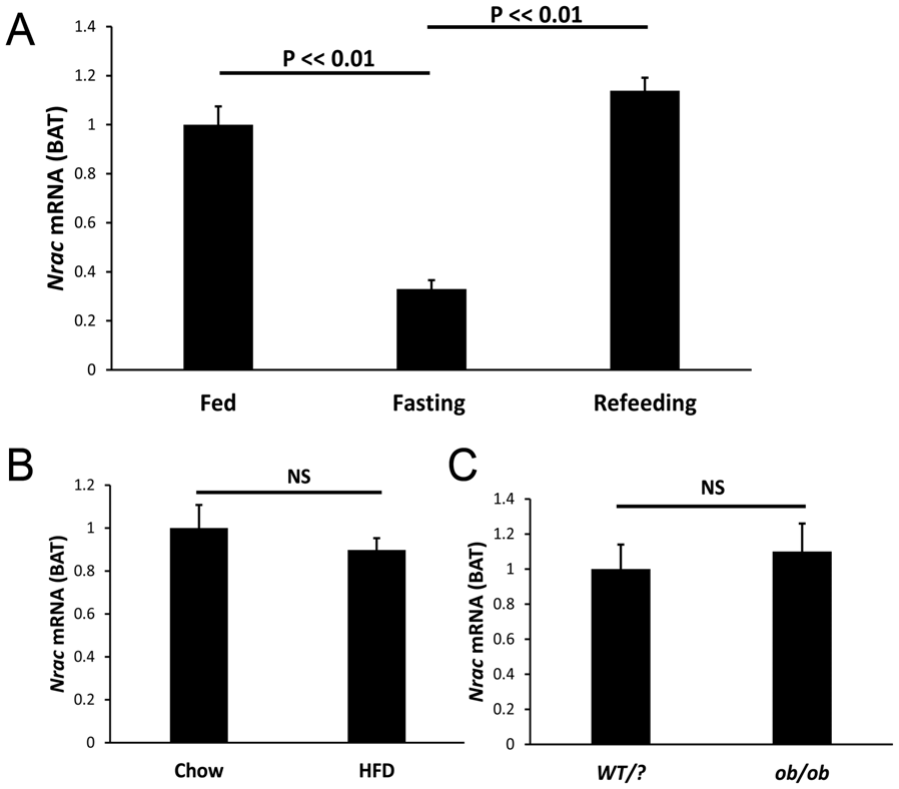
Nrac in brown adipose tissue (BAT) by nutritional stimulation. A) BAT *Nrac* is down regulated by 24-hour fasting, and normalized by 4-hour refeeding. B) BAT *Nrac* shows no significant change in diet induced obesity and C) in *ob*/*ob* mice. Data are represented as mean ± SEM.

To confirm *Nrac* is nutritionally regulated, we treated mice with fasting or HFD, and we also used the *ob/ob* mouse model, which lacks leptin, and then examined *Nrac* expression by qPCR analysis. In white adipose tissue (WAT), 24-hour fasting reduced *Nrac* expression for about 50% (P<0.01), and refeeding 4 hours following the fasting normalized its expression (Fig. 3A). In mice with 3-month of HFD treatment, *Nrac* expression in WAT was reduced for about 80% (P<<0.01) (Fig. 3B). Likewise, in *ob/ob* mice, WAT *Nrac* was also significantly reduced (P<0.01) (Fig. 3C). In brown fat, fasting reduced *Nrac* expression for about 70% (P<0.01), which was normalized by refeeding (Fig. 4A). Nevertheless, *Nrac* expression in BAT was not significantly changed in both diet induced obesity mice and *ob/ob* mice (Fig. 4B and C). Therefore, fasting reduces the expression of *Nrac* in both WAT and BAT, and obesity also reduces *Nrac* in WAT.

### Nrac is Localized to the Plasma Membrane

**Figure 5:**
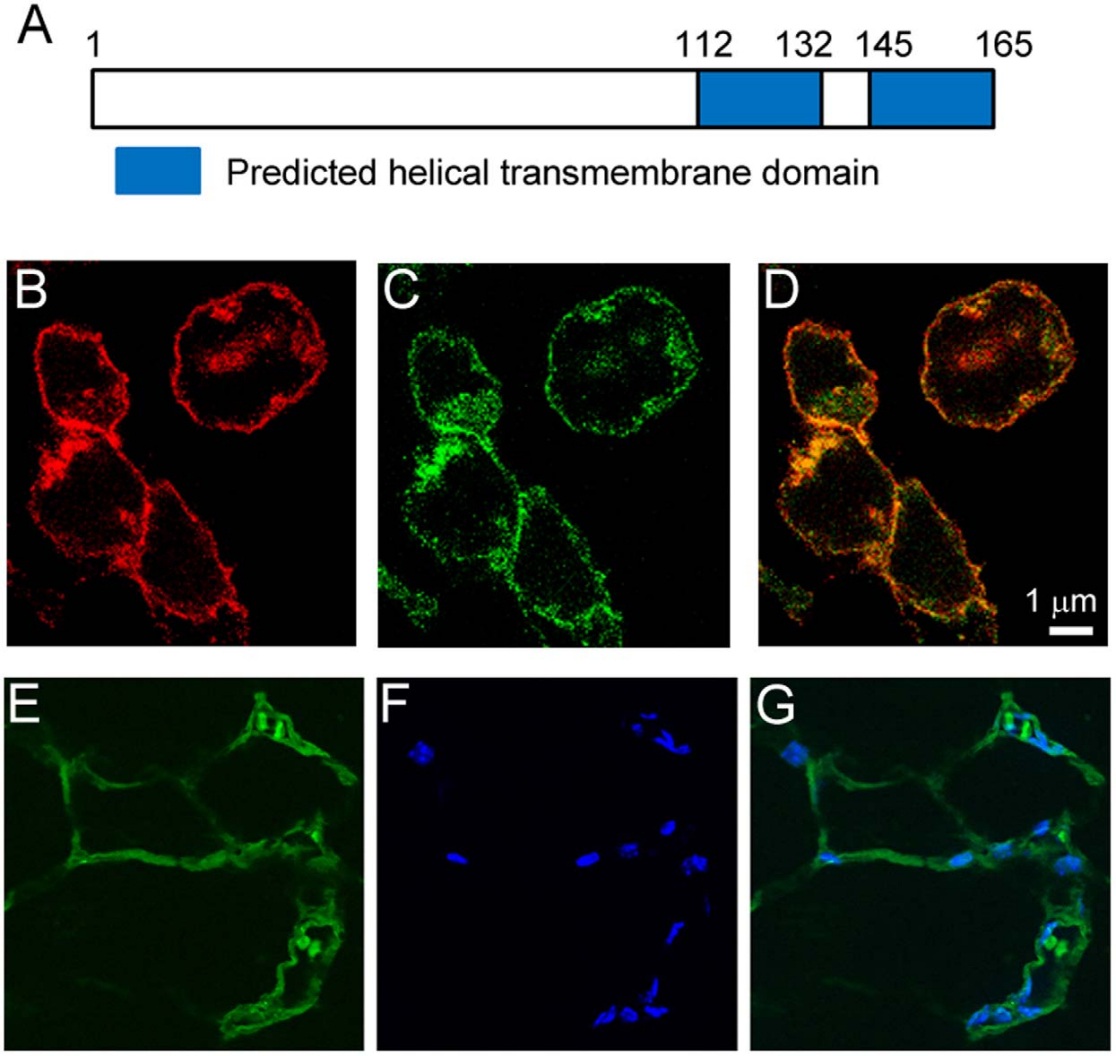
Nrac localizes to the plasma membrane. A) Predicted transmembrane domains. Fluorescence imaging of HEK293 cells transfected with plasmids encoding B) LCK-tRFP and C) Nrac-GFP and D) Merged picture. E) Immunostaining with a primary antibody against Nrac, visualized by an Alexa Fluor 488-conjugated secondary antibody, F) Hoechst staining for nuclei, and G) merged picture, in paraffin-embedded sections of mouse white adipose tissue.

Proteins need to localize to specific subcellular compartments to perform functions, therefore the subcellular localization of a protein is critical to reveal its functions. The Universal Protein Resource [25] annotates the protein as having 2 transmembrane domains, from amino acid 112 to 132, and from 145 to 165 (Fig. 5A). To gain experimental evidence, we examined the subcellular localization by fluorescent protein imaging. A fusion protein with a GFP at the C-terminal of Nrac was made by cloning the Nrac open reading frame into a vector containing the GFP gene. The vector encoding the fusion protein Nrac-GFP was then co-transfected with a vector encoding red fluorescent protein fused with LCK (lymphocyte-specific protein tyrosine kinase), which is localized to the plasma membrane. Indeed, red signals, which indicate the location of LCK, localized to the plasma membrane (Fig. 5B). Green signals, which indicate the location of Nrac, also localized to the plasma membrane (Fig. 5C). Merging of the two signals shows that Nrac and LCK co-localized (Fig. 5D), indicating that Nrac is localized to the plasma membrane. Because this result was obtained in HEK293 cells, the possibility that Nrac in present on the membrane of cellular organelles in other cell types should not be excluded. To further confirm Nrac subcellular localization, we performed Nrac immunostaining in paraffin-embedded sections of mouse white adipose tissue, and the primary antibody was visualized by an Alexa Fluor 488-conjugated secondary antibody (Fig. 5E–G). Indeed, consistent with results obtained in HEK293 cells, Nrac was localized to the plasma membrane of adipocytes.

### Nrac is Highly Induced During Adipocyte Differentiation

**Figure 6:**
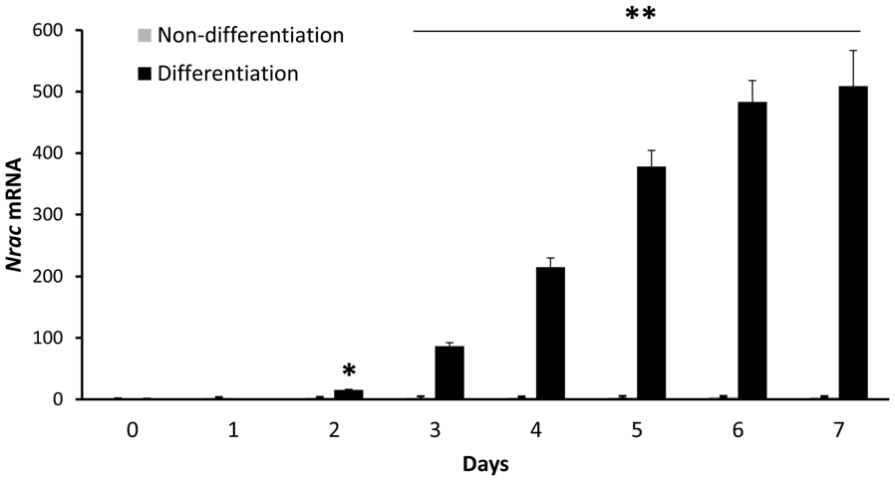
Nrac is highly induced during adipogenesis. 3T3 L1 preadipocytes were either differentiated into adipocytes or maintained in non-differentiation medium, and *Nrac* expression was determined by qPCR in cells before (day 0) and after (day 1 to 7) differentiation. Data are represented as mean ± SEM. *, P<0.05; **, P<0.01.

Adipogenesis is the process of cell differentiation by which preadipocytes become adipocytes. Because *Nrac* is highly expressed in WAT, we hypothesized that *Nrac* is induced during adipogenesis. To test the hypothesis, we differentiated 3T3 L1 preadipocytes into adipocytes, and examined *Nrac* expression. Two days after reaching confluence, cells were cultured in differentiation medium that contained insulin and PPARγ agonists for 3 days to induce differentiation, and after 3 days, regular maintenance medium was used for additional 4 days. Differentiation was confirmed by oil red O staining and by examining the expression of adipocyte marker genes, e.g., PPARγ (not shown). As a control, additional cells were also cultured in maintenance medium. In non-differentiation medium on the day 7, there was an about twofold increase in *Nrac* expression, in differentiation medium, however, *Nrac* was highly induced. On day 3, it was induced for more than 80-fold, and on day 7 (Fig. 6), it was increased for more than 500-fold. Therefore, *Nrac* is highly induced during adipogenesis.

Obesity increases the risk of numerous health consequences, such as insulin resistance, type 2 diabetes and coronary heart diseases, and therefore, the current obesity epidemic poses a major public health issue. Therapeutic approaches are urgently needed to treat obesity as well as its complications. Proteins presented on the cell surface are accessible to various drug delivery mechanisms, therefore plasma membrane proteins are attractive drug targets [26]. Another ideal characteristic for drug targets is tissue specificity. Drugs targeting genes with specific expression in certain tissues have the potential to alleviate side effects. In that regard, Nrac is a potentially attractive drug target because it is specific to fat and the heart, and localized to the plasma membrane.

The expression level of *Nrac* is sensitive to nutritional stimulation. Both fasting and obesity reduced *Nrac* expression in WAT. Because it is highly expressed in fat, and because it is highly induced during adipogenesis, it can be used as a novel adipocyte marker gene. Nrac is not only highly expressed in WAT; it is also abundant in brown fat. It is increasingly being recognized that adult humans have BAT as well, and its amount is inversely correlated with body mass index [27], [28]. It is possible that NRAC is also present in human brown fat and play a role in brown fat functions. The apparent next logical step would be to either overexpress or knock down *Nrac* expression in cells and mice to study its functions, and this line of work is being performed in this laboratory.

In summary, we have identified Nrac, a novel nutritionally regulated gene. *Nrac* is expressed specifically in fat and the heart. Being abundant in fat and highly induced during adipogenesis establish *Nrac* as a novel adipocyte marker gene. The functions of Nrac in white adipose tissue, brown fat as well as in the heart have yet to be explored.

## Materials and Methods

### Ethics Statement

All animal protocols were approved by the Animal Care and Use Committee of Wayne State University.

### Mice

Mice were housed at 22–24°C with a 14-h light, 10-h dark cycle and provided with ad libitum water and a chow diet (6% calories from fat, 8664; Harlan Teklad, Indianapolis, IN) unless otherwise indicated. To examine nutritional stimulation induced gene expression, 10 4-week-old male C57B6 mice (Jackson laboratory, Bar Habor, ME) were placed on either a chow diet or a high-fat, high-sucrose diet (58% kcal from fat, 26% kcal from sucrose, D-12331; Research Diets, New Brunswick, NJ) for 3 months. Five 8-week-old mice were treated with 24-hour fasting with 4 fed mice as controls. To examine the expression pattern of Nrac in various mouse tissues, 3 8-wk-old mice were used.

### RNA Extraction and Quantitative Real-time PCR

Dissected tissues were immediately placed into RNAlater solution (Ambion, Austin, TX) for subsequent RNA extraction. Total RNA was isolated from tissues with RNeasy tissue minikit with deoxyribonuclease treatment (QIAGEN, Valencia, CA). One microgram of RNA was reverse transcribed to cDNA using random hexamers (Superscript; Ambion). Relative expression levels were calculated and beta-actin was used as an internal control. Primer sequences for *Nrac* were: forward, 5′-TCTCTCGCTCTAATTCCCACC-3′; reverse, 5′-CACTTCCTGTTACCATCCCTCT-3′. Primer sequences for *β-actin* were: forward, 5′-GTGACGTTGACATCCGTAAAGA-3′; reverse, 5′-GCCGGACTCATCGTACTCC-3′.

### Multiple Alignments

Nrac orthologues and alignments were obtained from Ensembl Compara GeneTrees database using BioMart [29]. The names, taxonomically names and IDs for Nrac protein sequences are as follows: Panda, *Ailuropoda melanoleuca*, ENSAMEG00000008013; Cow, *Bos taurus*, ENSBTAG00000022775; Marmoset, *Callithrix jacchus*, ENSCJAG00000007889; Dog, *Canis lupus familiaris*, ENSCAFG00000018327; Guinea Pig, *Cavia porcellus*, ENSCPOG00000023615; Horse, *Equus caballus*, ENSECAG00000004388; Cat, *Felis catus*, ENSFCAG00000015032; Gorilla, *Gorilla gorilla*, ENSGGOG00000014934; Human, *Homo sapiens*, ENSG00000184601; Elephant, *Loxodonta africana*, ENSLAFG00000026667; Macaque, *Macaca mulatta*, ENSMMUG00000020194; Gibbon, *Nomascus leucogenys*, ENSNLEG00000016298; Pika, *Ochotona princeps*, ENSOPRG00000006160; Bushbaby, *Otolemur Garnettii*, ENSOGAG00000027809; Chimpanzee, *Pan troglodytes*, ENSPTRG00000006769; Orangutan, *Pongo abelii*, ENSPPYG00000006185; Rat, *Rattus norvegicus*, ENSRNOG00000013097; Tasmanian devil, *Sarcophilus harrisii*, ENSSHAG00000012200; Squirrel, *Spermophilus tridecemlineatus*, ENSSTOG00000020215; Tree Shrew, *Tupaia belangeri*, ENSTBEG00000011739 and Dolphin, *Tursiops truncatus*, ENSTTRG00000007344. DELTA-BLAST (Domain Enhanced Lookup Time Accelerated BLAST) is a program useful for detecting remote homologs [24]. In addition to the 21 orthologs in the Compara GeneTrees database [23], a search based on DELTA-BLAST using the human NRAC protein sequence against the NCBI protein database also showed significant alignments with LOC100556678 (*Anolis carolinensis,* Tilapia) and LOC100701668 (*Oreochromis niloticus,* lizard).

### Cell Culture and Imaging

3T3-L1 cells (American Type Cell Collection, Manassask, VA) were maintained in Dulbecco’s Modified Eagle Media (DMEM) containing 10% Fetal bovine serum (FBS) at 37°C with 5% CO_2_ in a humidified incubator. To differentiate 3T3-L1 into adipocytes, 48 hours after reaching confluence, preadipocytes (in 48-well plates with triplicates) were cultured in differentiation medium (Zenbio, Research Triangle Park, NC) for 3 days, followed by culturing the cells in maintenance medium for additional 4 days. To examine Nrac subcellular localization, fusion protein of Nrac-GFP was made by cloning the Nrac open reading frame (Origene, MD) into the pCMV6-AC-GFP vector. In HEK293 cells, the Nrac-GFP vector was cotransfected with a pCMV6 vector that encodes a fusion protein of LCK (lymphocyte-specific protein tyrosine kinase) and red fluorescence protein (Origene, MD). An antibody against Nrac was from Santa Cruz Biotechnology (Santa Cruz, CA), and the Nrac immunostaining with visualized by an Alexa Fluor 488-conjugated secondary antibody (Invitrogen, Grand Island, NY). Fluorescence Images were taken at the imaging core facility of Wayne State University with a Leica TCS SP5 Confocal Microscope using the software Zen blue edition from Carl Zeiss Microimaging Inc.

### Statistical Analysis

Data are expressed as the mean ± sem. Statistical significance was tested with unpaired two-tailed Student’s *t* tests. The differences were considered statistically significant if P<0.05.

## References

[1] WHO (2000) Obesity: preventing and managing the global epidemic. Report of a WHO consultation. World Health Organ Tech Rep Ser 894: i–xii, 1–253. 11234459

[2] Billington CJ, Epstein LH, Goodwin NJ, Hill JO, Pi-Sunyer JX, et al (2000) Overweight, obesity, and health risk. National Task Force on the Prevention and Treatment of Obesity. Arch Intern Med 160: 898–904. 10761953 10.1001/archinte.160.7.898

[3] Ogden CL, Carroll MD, Curtin LR, McDowell MA, Tabak CJ, et al (2006) Prevalence of overweight and obesity in the United States, 1999–2004. JAMA 295: 1549–1555. 16595758 10.1001/jama.295.13.1549

[4] Luo J, Hu FB (2002) Time trends of obesity in pre-school children in China from 1989 to 1997. Int J Obes Relat Metab Disord 26: 553–558. 12075583 10.1038/sj.ijo.0801944

[5] Rennie KL, Jebb SA (2005) Prevalence of obesity in Great Britain. Obes Rev 6: 11–12. 15655034 10.1111/j.1467-789X.2005.00164.x

[6] Wang Y, Monteiro C, Popkin BM (2002) Trends of obesity and underweight in older children and adolescents in the United States, Brazil, China, and Russia. Am J Clin Nutr 75: 971–977. 12036801 10.1093/ajcn/75.6.971

[7] Zhang Y, Proenca R, Maffei M, Barone M, Leopold L, et al (1994) Positional cloning of the mouse obese gene and its human homologue. Nature 372: 425–432. 7984236 10.1038/372425a0

[8] Spiegelman BM, Flier JS (2001) Obesity and the regulation of energy balance. Cell 104: 531–543. 11239410 10.1016/s0092-8674(01)00240-9

[9] Flier JS (2004) Obesity wars: molecular progress confronts an expanding epidemic. Cell 116: 337–350. 14744442 10.1016/s0092-8674(03)01081-x

[10] Schwartz MW, Woods SC, Porte D Jr, Seeley RJ, Baskin DG (2000) Central nervous system control of food intake. Nature 404: 661–671. 10766253 10.1038/35007534

[11] Barsh GS, Farooqi IS, O’Rahilly S (2000) Genetics of body-weight regulation. Nature 404: 644–651. 10766251 10.1038/35007519

[12] Rosen ED, Spiegelman BM (2000) Molecular regulation of adipogenesis. Annu Rev Cell Dev Biol 16: 145–171. 11031233 10.1146/annurev.cellbio.16.1.145

[13] Kershaw EE, Flier JS (2004) Adipose tissue as an endocrine organ. Journal of Clinical Endocrinology & Metabolism 89: 2548–2556. 15181022 10.1210/jc.2004-0395

[14] Ahima RS, Flier JS (2000) Adipose tissue as an endocrine organ. Trends in Endocrinology and Metabolism 11: 327–332. 10996528 10.1016/s1043-2760(00)00301-5

[15] Mohamed-Ali V, Pinkney JH, Coppack SW (1998) Adipose tissue as an endocrine and paracrine organ. International Journal of Obesity 22: 1145–1158. 9877249 10.1038/sj.ijo.0800770

[16] Trayhurn P, Beattie JH (2001) Physiological role of adipose tissue: white adipose tissue as an endocrine and secretory organ. Proceedings of the Nutrition Society 60: 329–339. 11681807 10.1079/pns200194

[17] Fruhbeck G, Gomez-Ambrosi J, Muruzabal FJ, Burrell MA (2001) The adipocyte: a model for integration of endocrine and metabolic signaling in energy metabolism regulation. American Journal of Physiology-Endocrinology and Metabolism 280: E827–E847. 11350765 10.1152/ajpendo.2001.280.6.E827

[18] Flier JS (1995) The Adipocyte - Storage Depot or Node on the Energy Information Superhighway. Cell 80: 15–18. 7813011 10.1016/0092-8674(95)90445-x

[19] Kennedy AR, Pissios P, Otu H, Xue BZ, Asakura K, et al (2007) A high-fat, ketogenic diet induces a unique metabolic state in mice. American Journal of Physiology-Endocrinology and Metabolism 292: E1724–E1739. 17299079 10.1152/ajpendo.00717.2006

[20] Xu HY, Barnes GT, Yang Q, Tan Q, Yang DS, et al (2003) Chronic inflammation in fat plays a crucial role in the development of obesity-related insulin resistance. Journal of Clinical Investigation 112: 1821–1830. 14679177 10.1172/JCI19451 PMC296998

[21] Weisberg SP, McCann D, Desai M, Rosenbaum M, Leibel RL, et al (2003) Obesity is associated with macrophage accumulation in adipose tissue. Journal of Clinical Investigation 112: 1796–1808. 14679176 10.1172/JCI19246 PMC296995

[22] Gupta RK, Rosen ED, Spiegelman BM (2011) Identifying Novel Transcriptional Components Controlling Energy Metabolism. Cell Metabolism 14: 739–745. 22152302 10.1016/j.cmet.2011.11.007 PMC3240865

[23] Vilella AJ, Severin J, Ureta-Vidal A, Heng L, Durbin R, et al (2009) EnsemblCompara GeneTrees: Complete, duplication-aware phylogenetic trees in vertebrates. Genome Research 19: 327–335. 19029536 10.1101/gr.073585.107 PMC2652215

[24] Boratyn GM, Schaffer AA, Agarwala R, Altschul SF, Lipman DJ, et al (2012) Domain enhanced lookup time accelerated BLAST. Biol Direct 7: 12. 22510480 10.1186/1745-6150-7-12 PMC3438057

[25] UniProtConsortium (2012) Reorganizing the protein space at the Universal Protein Resource (UniProt). Nucleic Acids Res 40: D71–75. 22102590 10.1093/nar/gkr981 PMC3245120

[26] Clark HF, Gurney AL, Abaya E, Baker K, Baldwin D, et al (2003) The secreted protein discovery initiative (SPDI), a large-scale effort to identify novel human secreted and transmembrane proteins: A bioinformatics assessment (vol 13, pg 2265, 2003). Genome Research 13: 2759–2759. 10.1101/gr.1293003 PMC403697 12975309

[27] Cypess AM, Lehman S, Williams G, Tal I, Rodman D, et al (2009) Identification and Importance of Brown Adipose Tissue in Adult Humans. New England Journal of Medicine 360: 1509–1517. 19357406 10.1056/NEJMoa0810780 PMC2859951

[28] Virtanen KA, Lidell ME, Orava J, Heglind M, Westergren R, et al (2009) Functional Brown Adipose Tissue in Healthy Adults (vol 360, pg 1518, 2009). New England Journal of Medicine 361: 1123–1123. 10.1056/NEJMoa0808949 19357407

[29] Guberman JM, Ai J, Arnaiz O, Baran J, Blake A, et al (2011) BioMart Central Portal: an open database network for the biological community. Database (Oxford) 2011: bar041. 21930507 10.1093/database/bar041 PMC3263598

